# Rapid detection of carriers with *BRCA1 *and *BRCA2 *mutations using high resolution melting analysis

**DOI:** 10.1186/1471-2407-8-59

**Published:** 2008-02-25

**Authors:** Elena A Takano, Gillian Mitchell, Stephen B Fox, Alexander Dobrovic

**Affiliations:** 1 Molecular Pathology Research and Development Laboratory, Department of Pathology, Peter MacCallum Cancer Centre, Locked Bag 1, A'Beckett St, Melbourne, Victoria 8006, Australia; 2 Familial Cancer Centre, Peter MacCallum Cancer Centre, Locked Bag 1, A'Beckett St, Melbourne, Victoria 8006, Australia; 3 Department of Pathology, University of Melbourne, Parkville, Victoria, 3010, Australia

## Abstract

**Background:**

Germline inactivating mutations in *BRCA1 *and *BRCA2 *underlie a major proportion of the inherited predisposition to breast and ovarian cancer. These mutations are usually detected by DNA sequencing. Cost-effective and rapid methods to screen for these mutations would enable the extension of mutation testing to a broader population. High resolution melting (HRM) analysis is a rapid screening methodology with very low false negative rates. We therefore evaluated the use of HRM as a mutation scanning tool using, as a proof of principle, the three recurrent BRCA1 and BRCA2 founder mutations in the Ashkenazi Jewish population in addition to other mutations that occur in the same regions.

**Methods:**

We designed PCR amplicons for HRM scanning of *BRCA1 *exons 2 and 20 (carrying the founder mutations185delAG and 5382insC respectively) and the part of the *BRCA2 *exon 11 carrying the 6174delT founder mutation. The analysis was performed on an HRM-enabled real time PCR machine.

**Results:**

We tested DNA from the peripheral blood of 29 individuals heterozygous for known mutations. All the Ashkenazi founder mutations were readily identified. Other mutations in each region that were also readily detected included the recently identified Greek founder mutation 5331G>A in exon 20 of *BRCA1*. Each mutation had a reproducible melting profile.

**Conclusion:**

HRM is a simple and rapid scanning method for known and unknown *BRCA1 *and *BRCA2 *germline mutations that can dramatically reduce the amount of sequencing required and reduce the turnaround time for mutation screening and testing. In some cases, such as tracking mutations through pedigrees, sequencing may only be necessary to confirm positive results. This methodology will allow for the economical screening of founder mutations not only in people of Ashkenazi Jewish ancestry but also in other populations with founder mutations such as Central and Eastern Europeans (*BRCA1 *5382insC) and Greek Europeans (*BRCA1 *5331G>A).

## Background

*BRCA1 *and *BRCA2 *are the two most frequently mutated genes underlying inherited breast and ovarian cancer. As both are large multi-exon genes, with inactivating mutations occurring across the entire coding region, the consequent cost of *BRCA1/2 *mutation screening has limited mutation testing to those who are most likely to have a mutation based on their family history of cancer. Consequently, a significant proportion of women carrying germline mutations are missing out on the opportunity to have mutation screening and thereby to modify their risk of breast cancer, particularly those with a small pedigree structure or where the inheritance is through the paternal lineage. The costs of full sequencing are reducing, but it will be some years before they drop significantly enough to have a serious impact on the numbers of genetic tests provided by scarce health dollars. In the meantime, an alternative approach is to use a mutation scanning technique to highlight variations in genomic sequences which are then characterised by sequencing.

High resolution melting (HRM) is a new methodology for mutation scanning in which the mutation scanning is carried out in the same tube or well in which the sequence is amplified (reviewed in [1]). The PCR reactions and HRM can all be performed in a single run of less than 2 hours resulting in extremely rapid screening. Up to 384 scans (generally 192 samples in duplicate) can be done at the same time enabling very high throughput.

We selected several regions of the *BRCA1 *and *BRCA2 *genes known to carry a number of founder mutations in order to investigate the ability of HRM for reliable detection of both known founder mutations and other mutations occurring in close proximity to those founder mutations. Founder mutations are specific mutations found at high frequency in a particular population as a result of geographic, cultural or ethnic isolation. Individuals of Ashkenazi Jewish ancestry have a particularly high carrier rate for three mutations predisposing to the hereditary breast and ovarian cancer syndrome: the 185delAG and the 5382insC in *BRCA1 *and the 6174delT in *BRCA2 *with a population prevalence of approximately 2.5% [2-4]. The 5382insC in exon 20 of *BRCA1 *is also one of the most common recurrent mutations in Central and Eastern Europeans [5-9]. This exon also contains the 5331G>A BRCA1 mutation recently shown to be a founder mutation in Greek Europeans [10].

## Methods

### Samples

Samples from 29 carriers of known mutations in one of the regions of the *BRCA1 *and *BRCA2 *genes that contain the Ashkenazi founder mutations were obtained from existing samples in the Diagnostic Molecular Pathology laboratory at the Peter MacCallum Cancer Centre. Up to five biological replicate samples (i.e separate individuals) were chosen for each mutation. The study was performed under guidelines approved by the Peter MacCallum Ethics of Human Research Committee (approval 03/90).

### HRM Assay Conditions

Effective primer design is an important component of HRM analysis. Primers were designed to flank the coding regions of *BRCA1 *exons 2 and 20 and to amplify a 534 bp region surrounding the 6174delT mutation on *BRCA2 *exon 11. All amplicons were chosen so that they contained a single melting domain using the Poland program [11]. It was also necessary to ensure that the primers did not overlay known single nucleotide polymorphisms (SNPs) that may lead to single alleles not being amplified. An example of this was previously reported for the 6174delT mutation where the wild type allele was not amplified[12]. In other cases, this would lead to lack of amplification of the mutant allele. The primers were designed to be annealed at 60°C using Primer Express software (Applied Biosystems, Foster City, CA) to calculate the "Tm". Primer sequences are listed in Table 1.

**Table 1:** List of primers used to amplify *BRCA1 *exon 2, *BRCA1 *exon 20 and part of *BRCA2 *exon11.

**Gene**		**Sequence**
***BRCA1***	Forward primer	5'-AAAAGATATAGATGTATGTTTTGCTAATGTGT-3'
**exon 2**	Reverse primer	5'-TCCCAAATTAATACACTCTTGTGCTGA-3'
***BRCA1***	Forward primer	5'-GAGTGGTGGGGTGAGATTTTTGTC-3'
**exon 20**	Reverse primer	5'-CCTGATGGGTTGTGTTTGGTTTCT-3'
***BRCA2***	Forward primer	5'-CGAAAATTATGGCAGGTTGTTACG-3'
**exon 11**	Reverse primer	5'-GCTTTCCACTTGCTGTACTAAATCCA-3'

### PCR and HRM Assay Conditions

The PCR and HRM were performed in a single run on a LightCycler 480 (Roche Diagnostics, Penzberg, Germany) in a reaction mix containing 2.5 μl (25 ng) of genomic DNA, either 400 nM (exon 2 and exon 11) or 200 nM (exon 20) of each primer and 3 mM MgCl_2 _in the LightCycler^® ^480 High Resolution Melting Master containing ResoLight dye (Roche Diagnostics) with PCR grade water adjusted to a total volume of 10 μl. The reaction condition included an activation step at 95°C for 10 minutes followed by 55 cycles of 95°C for 10 seconds, a touch down of 65°C to 55°C for 10 seconds (1°C/cycle) and 72°C for 30 seconds. Before the high resolution melting step the products were heated to 95°C for 1 minute. The HRM was carried out over the range from 72°C to 95°C rising at 1°C per second with 30 acquisitions per degree. All reactions were performed in duplicate in a 96-well microtiter plate.

### HRM Analysis

Upon completion of the run (approximately 2 hours), HRM curve analysis was performed using the LightCycler 480 Software version 1.3. supplied with the LightCycler 480. The melting curves were normalised and temperature shifted to allow samples to be directly compared. Difference plots were generated by selecting a negative control, converting the melting profile to a horizontal line and normalising the melting profiles of the other samples against this sample Significant differences in fluorescence from the horizontal baseline were indicative of mutations. Differences were judged as significant if the replicates fell outside the range of variation seen in the wild type samples.

## Results

We tested DNA from the peripheral blood of individuals heterozygous for known mutations in the three regions containing the Ashkenazi founder mutations predisposing to breast and ovarian cancer as well a series of wild type controls (Table 2). As in our previous publications on mutation detection [13,14], we found that difference curves, where the melting profile of one of the control samples is converted to a horizontal line and the melting profiles of the other samples are normalised against this line, allowed the best visualisation of mutations.

**Table 2:** List of mutations tested

***BRCA1 *exon 2**		***BRCA1 *exon 20**		***BRCA2 *exon 11**	
Mutation	Number of samples	Mutation	Number of samples	Mutation	Number of samples
Wild type	13	Wild type	16	Wild type	26
188del11	1	5396+1G>A	4	6174delT	3
185delAG	4	5382insC	6	6293C>G	2
185insA	2	5331G>A	5	6024delTA	2

**Figure 1:**
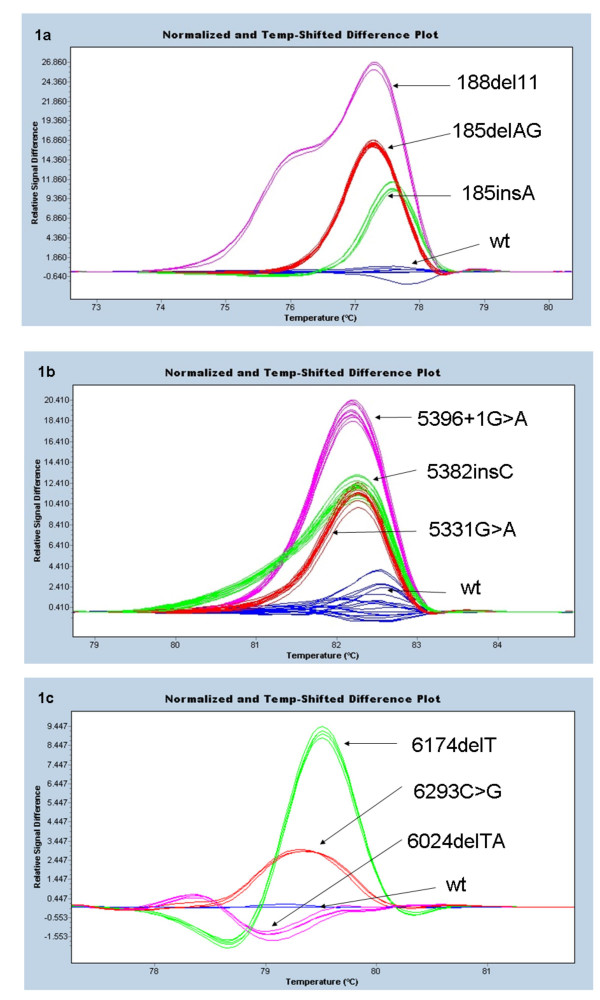
**Difference plot showing different mutations relative to the wild type controls**. In difference plots, the melting profile of a wild type control is chosen as a horizontal base line and the relative differences in the melting of all the other samples are plotted relative to this baseline. The figure shows difference plots for each of the 3 exons containing one of the common Ashkenazi mutations (185delAG, 5382insC and 6174delT). Each trace represents the amplicon from a different individual's DNA sample. All mutations were clearly distinct from the wild type controls. a. *BRCA1 *exon 2: Melt curves of each mutation (pink: 188del11, red: 185delAG and green: 185insA) were plotted against the wild type (blue). b. *BRCA1 *exon 20: Melt curves of each mutation (pink: 5396+1G>A, red: 5331G>A and green: 5382insC) were plotted against melt curve of the wild type (blue). **c**. *BRCA2 *exon 11: Melt curves of each mutation (pink: 6024delTA, red: 6293C>G and green: 6174delT) were plotted against melt curve of the wild type (blue).

For *BRCA1 *exon 2 (Figure 1a), we tested DNA from 4 individuals with the Ashkenazi mutation 185delAG as well as 1 individual with the mutation 188del11 and 2 individuals with the mutation 185insA. The mutations were all readily differentiated from the wild type. They were also readily differentiated from each other giving characteristic melting curves as is readily seen from the figure. It can also readily be seen that biological replicates had near identical melting patterns.

Similarly for the amplicon including *BRCA1 *exon 20 (Figure 1b) and for the amplicon including the region around the 6174delT mutation in BRCA2 exon 11 (Figure 1c), all the mutations were readily identified, displayed characteristic melting curves and were clearly differentiated from the wild type and from each other. The mutations tested in addition to the 5382insC (6 individuals) were the 5396+1G>A (4 individuals) and the 5331G>A (5 individuals). The mutations tested in addition to the 6174delT (3 individuals) were the 6024delTA (2 individuals) and the 6293C>G (2 individuals). Biological replicates again had near identical melting patterns.

## Discussion

DNA sequencing is regarded as the gold standard for *BRCA1 *and *BRCA2 *mutation detection. However, in practice, most testing laboratories perform a preliminary scan of exons and splice junctions using methods such as the protein truncation test (PTT), denaturing gradient gel electrophoresis (DGGE) and/or denaturing high pressure liquid chromatography (DHPLC) to reduce cost and increase throughput. Any region that is found to potentially contain a mutation by scanning is then characterised by sequencing.

Scanning methods are particularly relevant to studies involving large numbers of individuals. A recent Canadian study [15] used a combination of PTT, DGGE and DHPLC to scan the *BRCA1 *and *BRCA2 *genes, followed by DNA sequencing to screen for *BRCA1 *and *BRCA2 *mutations. Although very sensitive, PTT, DGGE and DHPLC do not detect all mutations. In particular, PTT, which is used for screening of the large exons comprising approximately half of the total sequence of the *BRCA1 *and *BRCA2 *genes, does not detect missense alterations. Both DHPLC and DGGE are both operator and interpreter sensitive. It has been shown that DHPLC, which is currently considered as the gold standard of screening methods, is superior to DGGE for the detection of *BRCA1 *and *BRCA2 *mutations [16].

HRM is an in-well scanning method in which the melting analysis is performed immediately after the PCR amplification and is thus particularly suitable for high-throughput applications. HRM is predicted to have close to a 100% sensitivity as each heterozygous mutation should create a readily detected heteroduplex [17]. Heterozygotes are readily recognisable by HRM because they form a proportion of heteroduplexes which melt differently to perfectly matched homoduplexes. The use of HRM to detect both known and novel germline mutations has been reported for several other genes including the *EXT1*, *EXT2*, *RET*, *GJB1 *and *CFTR *genes [18-21]. All germline mutations in the *BRCA1 *and *BRCA2 *genes are expected to occur in heterozygotes as mutant homozygotes are lethal [22] and consistent with this, none have been reported.

A comparative study has recently shown that the sensitivity and specificity of HRM is better than DHPLC which is the current gold standard of scanning methods [17]. In addition, HRM is more rapid as the melting analysis is performed in all wells simultaneously whereas DHPLC involves the removal of the PCR product (in itself problematical because of the PCR contamination issue) and then the sequential analysis of each sample. The instrumentation for DHPLC is more expensive and the analysis generates chemical waste which requires disposal. There are therefore significant time and cost advantages in adopting HRM.

In our hands, all the different mutations in the 3 regions that we analysed from the *BRCA1 *and *BRCA2 *genes were detected, giving us a sensitivity of 100% in this pilot study. The true sensitivity remains to be determined in a larger study, but publications for other genes indicate that it is likely to be very high [14,17]. A larger study is currently underway in our lab testing several hundred known mutations in historic samples across all *BRCA1 *and *BRCA2 *coding regions in combination with the screening of prospectively collected diagnostic samples. This will provide further insight into the true sensitivity of HRM and applicability of the method for mutation scanning of the entire coding regions of *BRCA1 *and *BRCA2*.

Currently, the three founder mutations associated with Ashkenazi Jewish ancestry are generally identified using sequencing and, once a mutation is identified in a family, other family members can undergo predictive testing by sequencing for the exon in which the mutation was identified. However, as the frequency of the three founder mutations is relatively high, it is possible that a second mutation might be segregating in the family and so ideally all founder mutations should be sought in other family members. HRM would allow testing for all three Ashkenazi mutations in family members at relatively low cost. The testing would also enable inexpensive population-based screening of individuals with Jewish ancestry for the founder mutations.

We also detected the 5331G>A mutation in *BRCA1 *exon 20 that has recently been shown to be a Greek founder mutation [10]. Large scale screening for this founder mutation can now be undertaken in relevant populations. A similar approach can be applied to other founder mutations in regions not screened for in this study. The ability to detect mutations by HRM alone has implications for economical large scale population screening studies to understand the prevalence and characteristics of other known or suspected founder mutations.

HRM can also be used as an inexpensive methodology for tracking mutations across pedigrees. Each mutation had a characteristic melting profile that would allow it to be identified by using a control for that mutation. In some cases, this would remove the necessity for sequencing. However, for diagnostic purposes, any apparently mutation positive individual should be confirmed by sequencing as different mutations can also have similar profiles.

Dufresne and colleagues [23] reported the use of melt curve analysis for the specific detection of the Ashkenazi mutations using SYBR Green 1 incorporation as monitored by a standard real time PCR machine. This approach is melting analysis rather then the high resolution melting (HRM) we have used here. Whereas their methodology is capable of detecting known mutations such as the Ashkenazi mutations by designing primers flanking those mutations, our methodology is an essentially different and more powerful methodology which is designed primarily as a screening methodology. We used an appropriate dye for high resolution melting, an HRM enabled real time PCR machine (only recently available) and software that allows HRM analysis to be performed. The greater sensitivity of HRM allowed longer amplicons to be used (534 base pairs was successfully used for the region surrounding the 6174delT mutation) which allows for the screening of unknown mutations as we have demonstrated. Thus by examining extra, non-founder mutations in close proximity to the founder mutations we show that this method easily detects missense mutations as well as small insertions and deletions. Interestingly, the missense mutation heterozygotes melt earlier than some of the deletion/insertion heterozygotes; the 6174delT initially being more stable than even the wildtype.

Based on our current findings, we are confident that HRM for mutation detection will prove superior to the current germline mutation screening approaches in terms of speed, and cost and give at least equivalent sensitivity. HRM is the most rapid of the current screening methods as the only set-up involved is the PCR amplification, up to 384 reactions can be tested in the one run for a given amplicon and the results can be rapidly scanned by a technician. Consequently, HRM is the least expensive of the current screening methods as the only consumables cost involved is the PCR amplification with a HRM dye and the reaction vessels while the rapid scanning reduces labour costs. Consequently, we believe that HRM may become the method of choice for *BRCA1 *and *BRCA2 *mutation screening in both the diagnostic and research settings.

## Conclusion

This study demonstrates the use of HRM as a method to scan for the common germline *BRCA1 *and *BRCA2 *mutations such as those seen in Ashkenazi Jewish individuals. It also demonstrates the potential of this method for screening the *BRCA1 *and *BRCA2 *genes as every mutation tested for was readily detected. Whereas, the use of HRM for full screening of the *BRCA1 *and *BRCA2 *still needs to be verified by sequencing, screening for the Ashkenazi founder mutations (or other founder mutations) can now be carried out by laboratories with access to the increasingly available high resolution melting platform. The method is sensitive, rapid and cost effective and will markedly reduce the amount of sequencing required in mutational studies of *BRCA1 *and *BRCA2*, and thereby the time and cost required for these studies.

## Competing interests

AD received a honorarium for a talk at a Roche Diagnostics symposium in 2007. All other authors declare no competing interests

## Authors' contributions

ET carried out the HRM studies, prepared the figures and assisted with the manuscript. AD was responsible for primer design and data analysis, and prepared the manuscript. SF and GM co-wrote the manuscript. All authors read and approved the final manuscript.

## Pre-publication history

The pre-publication history for this paper can be accessed here:


